# Reverse Δ-wave as a possible sign in electrocardiography to diagnose mitral valve prolapse

**DOI:** 10.1186/1756-0500-4-16

**Published:** 2011-01-25

**Authors:** Azin Alizadeh-Asl

**Affiliations:** 1 Cardiovascular Research Center, Tabriz University of Medical Sciences, Tabriz, Iran

## Abstract

**Background:**

Mitral valve prolapse (MVP) is defined as superior displacement of the mitral valve leaflets more than 2 mm into the left atrium during systole. Easier and cheaper assessment of this common disease is a priority in cardiac health care facilities.

**Presentation of the hypothesis:**

In this study I addressed electrocardiographic presentation in 300 patients with MVP compared with 100 healthy individuals. I faced a novel finding in electrocardiogram (ECG) examination of these patients. It was a notch (reverse Δ-wave) in descending arm of QRS observed in 79% (237/300) of patients, consisting of 58% (174/300) in inferior leads and 21% (63/300) in I and aVL leads. The notch was identified only in 6 men in control group.

**Testing the hypothesis:**

Considering the relatively higher prevalence of disease, a population-based diagnostic clinical trial study is appropriate to test the hypothesis.

**Implications of the hypothesis:**

The hypothesis on diagnostic value of reverse Δ-wave in MVP may help in decreasing the rate of unnessessary echocardiography in some patients.

## Background

Mitral valve prolapse (MVP) is defined as superior displacement of the mitral valve leaflets more than 2 mm into the left atrium during systole [1]. MVP is the most common primary valvular abnormality in a young population [1,2]. "MVP syndrome" is a term often used to describe a constellation of mitral valve prolapse and associated symptoms like palpitation or other physical abnormalities such as autonomic dysfunction and pectus excavatum [1]. In some individuals, MVP is silent or associated with palpitations, dizziness, chest pain, abnormal electrocardiogram (ECG) findings and sometimes serious complications [2-4].

### Presentation the hypothesis

In a prospective observational study, 300 (192 female and 108 male) patients with average age of 29 ± 8.5 years and MVP syndrome using strict echo criterion referred to cardiology clinics in Tabriz University of Medical Sciences from May 2007 to June 2009 were studied. The exclusion criteria were: Associated cardiac disorders ,being resuscitated after idiopathic ventricular fibrillation, a history of unexplained syncope and a familial incidence of sudden death at a young age.

**Figure 1:**
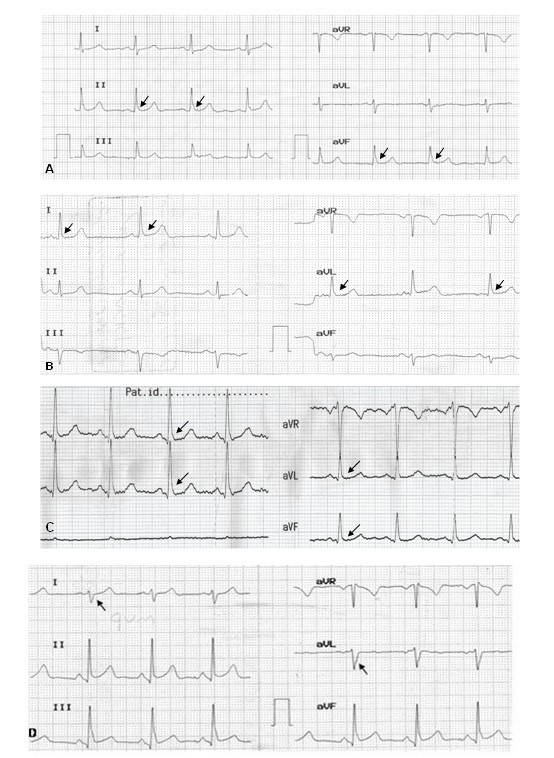
**Electrocardiographic changes in mitral valve prolapse**. *A: Reverse Δ-wave in inferior leads. B: Reverse Δ-wave in I and aVL leads. C: Reverse Δ-wave in both inferior, I and aVL leads. D: rS in I and aVL leads with reverse Δ-wave in inferior leads.*

The age-sex matched control group included 100 healthy individuals (58 female and 42 male, mean age: 31 ± 6.9 years) without any complaints and who had normal echocardiography. I faced a new finding in ECG examination of MVP patients; it was a notch (reverse Δ-wave) in descending arm of QRS observed in 79% ( 237/300) of patients; consisting of 58% (174/300) in inferior leads (Figure 1A) and 21% (63/300) in I and aVL leads (Figure 1B) and occasionally in both [6.3% (15/237)]; Figure 1C); whereas this notch was only seen in six men in control group.

**Figure 2:**
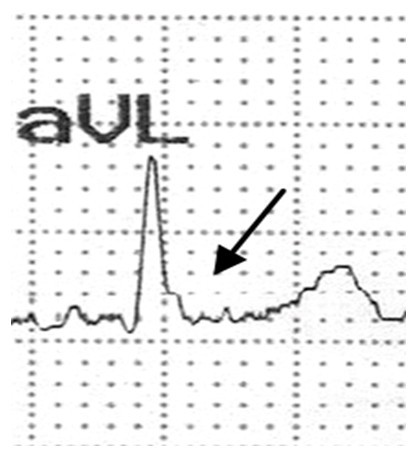
**A close up picture of the reverse Δ-wave in aVL lead**.

In addition, this sign was commonly seen (91.1%) in descending arm of QRS and rarely (8.9%) appeared immediately after QRS. Other common ECG changes in the remaining patients (although sometimes in the above mentioned patients) were Rs or rS in inferior or in I and aVL leads (Figure 1D). A close up view of the reverse Δ-wave in aVL lead is provided in figure 2.

Majority (202/237, 85.2%) of our patients with this sign had thin mitral valve leaflets. However, regarding the presence of the reverse Δ-wave in remaining 14.8%, it seems this sign is independent of leflet thickness and also presence of mitral regurgitation.

### Testing the hypothesis

Our methodology suffers some limitations that our findings can't be considered as confirmatory study results. So we presented it as a hypothesis which needs to be tested in a proper way. To test the hypothesis considering the relatively higher prevalence of disease, a population based diagnostic clinical trial study needs to be conducted. In such a study this ECG finding will be checked in eligible general population and compared to the combined results of echocardiography and clinical examination as gold standard. This will help to assess the accuracy and to test weather existence of this notch assures a given level of sensitivity and specificity or not. To increase precision in testing the hypothesis, supervised modeling of numerical data may also be used in conjunction with graphical assessment of ECG.

### Implications of the hypothesis

If future focused studies confirm our hypothesis, it will help to decreases the rate of unnessessary echocardiography in some patients. As ECG is available wider than echocardiography and can easily used and interpreted even by general practitioners increasing the general access to diagnostic facilities.

## Competing interests

The authors declare they have no competing interests.

## Authors' contributions

A to Z of this research is done by the sole author of the manuscript.
